# Irresponsibly Infertile? Obesity, Efficiency, and Exclusion from Treatment

**DOI:** 10.1007/s10728-019-00366-w

**Published:** 2019-03-08

**Authors:** Rebecca C. H. Brown

**Affiliations:** Oxford Uehiro Centre for Practical Ethics, University of Oxford, Suite 8 Littlegate House, 16-17 St Ebbes Street, OX1 1PT Oxford, UK

**Keywords:** Health policy, Public health ethics, Infertility, Subfertility, Moral responsibility

## Abstract

Many countries tightly ration access to publicly funded fertility treatments such as in vitro fertilisation (IVF). One basis for excluding people from access to IVF is their body mass index. In this paper, I consider a number of potential justifications for such a policy, based on claims about effectiveness and cost-efficiency, and reject these as unsupported by available evidence. I consider an alternative justification: that those whose subfertility results from avoidable behaviours for which they are responsible are less deserving of treatment. I ultimately stop short of endorsing or rejecting such a justification, though highlight some reasons for thinking it is unlikely to be practicable.

## Introduction

There is considerable public, political and philosophical debate about the provision of assisted reproductive technologies (ART) such as in vitro fertilisation (IVF), particularly where these services are to be funded through state supported healthcare systems and general taxation [27, 29]. In this paper I consider the exclusion from fertility treatment of obese people. I discuss a number of potential justifications for a policy of refusing publicly funded IVF provision to obese people, and argue that the reasons healthcare providers claim motivate such a policy do not do the justificatory work required. I offer an alternative explanation (note: not *justification*) for such policies, relating to the responsibility obese people may bear for their subfertility. I consider how we might begin to explore whether it would be appropriate to limit IVF access for those who are obese on the basis of responsibility, and highlight some problems with such an approach to healthcare provision.

For the purposes of this discussion I shall largely focus on the United Kingdom’s healthcare system as an example. The British system lends itself to assessment because of its quasi-centralised structure. It has explicit methods for addressing issues of resource distribution centrally (through the National Institute for Health and Care Excellence, NICE), with final decisions about service provision at the regional level made by local commissioners (clinical commissioning groups [CCGs] in England, health boards in Scotland, Wales and Northern Ireland). However, much of the discussion is relevant to other national contexts where decisions about equitable provision of healthcare must be addressed.

## Policy Guidance and Exclusion of People with Obesity

In the UK, guidance about what treatments and services should be provided through the NHS is produced by NICE. The role of NICE is to synthesise relevant evidence and consider whether particular treatments provided to particular patient populations will provide sufficient health gains, given their cost, to make their funding justifiable, given the other demands placed on healthcare resources. I will discuss further in a later section the procedures NICE uses for this purpose. The guidance that NICE provides is *advisory*: final decisions about what treatment will be provided and to whom are determined by local commissioners.

Relating to subfertility and the provision of IVF, NICE recommends that local commissioners should offer three cycles of IVF to women under the age of 40 who have unexplained subfertility and have not conceived after 2 years of trying through regular, unprotected, heterosexual intercourse (NICE 2013). The guidelines recommend that women aged 40–42 should be offered one cycle of IVF. The recommendations for specific diagnosable causes of subfertility differ from those relating to unexplained subfertility, which is reported to affect somewhere between 10 and 40% of couples with subfertility [3, 23, 37]. For much of this paper I will be concerned with treatment for unexplained subfertility, for which IVF is the main available intervention.

NICE provide further guidance relating to alcohol consumption, smoking, caffeinated beverages, obesity, low body weight, tight underwear, occupation, prescribed, over-the-counter and recreational drug use, complementary therapy and folic acid supplementation. Some of these factors are thought to affect fertility in various ways, and can influence the way treatment is provided. I will focus on obesity here, but it could be that some of these other factors warrant similar scrutiny.

Regarding obesity, NICE state:

Women who have a body mass index (BMI) of 30 or over should be informed that they are likely to take longer to conceive…

Women who have a BMI of 30 or over and who are not ovulating should be informed that losing weight is likely to increase their chance of conception…

Women should be informed that participating in a group programme involving exercise and dietary advice leads to more pregnancies than weight loss advice alone…

Men who have a BMI of 30 or over should be informed that they are likely to have reduced fertility…

[34] pp. 15–16.

It is worth noting that NICE guidelines do not explicitly advise against providing IVF to women with obesity, they only flag that obesity may impact fertility and recommend providing certain weight loss advice to those women. A report written by the National Infertility Group for Scotland, however, does make specific recommendations that women with a BMI over 30 should not be offered fertility treatment [30].

In fact, commissioners responsible for determining what NHS services will be available for local populations invariably impose a BMI limit for treatment, and independent units which provide fertility services both privately and for the NHS also often impose a BMI limit of their own [59]. The BMI limit is imposed by almost all CCGs/health boards and generally requires that women’s BMI be 19 or over and under 30 (England, Scotland, Wales) or 35 (Northern Ireland), often for at least 6 months prior to receiving IVF [9, 10].^1^ In addition, some commissioners impose a BMI limit on the male partner of a couple wishing to access IVF [6]. My focus in this paper will be on the upper BMI limit as applied to women; similar criticisms are likely to be even more applicable to restrictions placed on male BMI, but I set these aside, along with the lower BMI limit placed on women seeking to access IVF.

The approach in the UK is comparable to a number of overseas contexts. The Royal Australian and New Zealand College of Obstetricians and Gynaecologists have published guidance that a BMI over 35 should be regarded as an absolute contraindication for IVF [56, 57]. In the absence of professional body guidelines, a 2016 survey of providers of fertility services in the United States found that the majority of responding centres have a formal policy of setting a maximum BMI threshold for IVF [18]. A draft report from the Canadian Fertility and Andrology Society does not recommend imposing a BMI cutoff, though notes that there is significant appetite for this, and that a 2014 survey of Canadian medical directors found that 50% of respondents imposed a cut-off [7, 24].

What is the justification for imposing an upper BMI limit on women seeking to access IVF services? In the following sections I will consider a number of bases for deeming it appropriate to exclude obese people from NHS-funded IVF.

## Proposed Justification I: IVF is Futile for Obese Women

One basis for refusing IVF to obese women could be that it confers no clinical benefit. Medical futility is a contested concept, insofar as judgements of futility often differ between different groups (physicians, patients, funders, philosophers) as well as between individuals. This is significantly due to the fact that these different groups may value different things, or be willing to tolerate different degrees of risk, side-effects, benefits, etc. in deciding what treatments count as worthwhile.

Without fully specifying what will constitute futile treatment in the case of IVF, we might specify some parameters. This will include, most clearly, treatment that is known with certainty will not result in the birth of a living child.^2^ It is likely that we should extend this to include treatment that is vanishingly unlikely to result in the birth of a living child. In addition to considering the rarity of benefits conferred, we should also consider the costs associated with the treatment, and begin to incorporate these into an assessment of whether or not IVF is likely to be of clinical benefit. As well as considerable financial costs, IVF is associated with a number of (risks of) harms. One such risk is developing ovarian hyperstimulation syndrome, which can result from taking drugs to stimulate egg cell production and in rare cases can be fatal. Other procedures associated with IVF, as well as the risks associated with pregnancy and childbirth in general, should be considered as detracting from the net (potential) benefit of IVF. Thus, it might be appropriate to refuse treatment where there is a non-zero chance of a live birth resulting, but a reasonably high risk of serious side-effects, such that the net effect is very likely to be negative.

Obesity is a risk factor for subfertility, and obese pregnancies are associated with higher risk of complications that threaten the health of the mother as well as reduce the likelihood of a live birth and potentially the longer term health of the child born [24, 34, 51]. However, it seems unlikely to be appropriate to describe IVF treatment as futile in obese women but not non-obese women: as described by Tremellen et al. [57], analysis of a large sample of cycles in North America showed live birth rates in morbidly obese women (those with a BMI over 35) were not much lower than in women in the healthy weight range (26.3% as opposed to 31.4%).

## Proposed Justification II: IVF is Insufficiently Cost-Effective

On any healthcare system there are likely to be strong objections to the provision of medically futile treatment. I have shown that current evidence suggests IVF is not necessarily futile in obese women (although there may be obese women for whom it is futile) and so this does not seem an appropriate justification for excluding them from treatment. For a nationalised healthcare system such as the NHS, the need to fairly distribute finite resources requires that justifiable treatments are not only not futile, but that they be sufficiently cost-effective. In this section I will summarise how cost-efficiency is judged within the UK healthcare system, and argue that it is inconsistent to judge IVF provision for obese women to be cost-inefficient whilst judging IVF provision for non-obese women to be cost-efficient.

Rather than showing that IVF in obese women *is* cost-effective, my claim here will be that the procedures used to judge cost-effectiveness in the context of IVF are inadequate, and that there is probably insufficient evidence at present to make a reliable judgement regarding the cost-effectiveness of IVF in either obese or non-obese women.

The gold standard of evidence in evidence-based medicine is the randomised controlled trial (RCT). This takes a population of people, allocates them at random to either the treatment (in this case, receiving IVF) or control group (either no treatment at all, placebo treatment, or some alternative treatment) and compares the effects observed in the treatment arm with those in the control arm. The significance of RCTs is that, if well designed and executed, they provide a reliable way of assessing the causal effects of a treatment. In the context of IVF this is particularly important because of the possibility of spontaneous conception, which I will say more about.

Subfertility is typically defined by failure to conceive following a period of regular, unprotected, heterosexual intercourse (normally at least a year) (see [33, 34, 60]). Subfertility can also be diagnosed in the presence of a clear subfertility-causing factor, such as where the individual is known to have undergone sterilisation or lacks viable gametes. A diagnosis of subfertility according to the one-year-without-conceiving definition does not indicate that a couple *cannot* conceive without assistance, but rather, it indicates that they have lower fertility than those who conceive within a year. Hence why I (and others) prefer the term *sub*fertility to *in*fertility, since the latter is erroneously suggestive of sterility.

The most likely time to conceive is within the first month of trying. With each subsequent month conception becomes increasingly less likely. Within the first year of trying, 80% of couples will conceive through intercourse; of the 20% who do not conceive in the first year of trying, 50% will conceive during the second year of trying [38]. This trend continues, with decreasing numbers of people conceiving each subsequent year [14, 55]. This means that if the population of people who did not conceive after 2 years of trying were all provided with IVF, some of those who successfully conceive would have conceived anyway in the absence of IVF. RCTs allow us to estimate what *additional* proportion of people conceive in the presence of IVF, and this is particularly important in the context of unexplained subfertility.

There is, however, very little good quality evidence relating to the effectiveness of IVF in people with unexplained subfertility. A Cochrane review^3^ published in 2012 identified just six RCTs looking at the effectiveness of IVF for treating unexplained subfertility [42]. The authors concluded that the paucity of data meant it was not possible to establish how effective IVF is. In fact, only two trials compared IVF to expectant management (i.e. not receiving any treatment and continuing to try to conceive through intercourse). Of these, one used live birth rates as the outcome measure [16], whilst the other used pregnancy rate [52]. The former reported a strong effect of IVF and the latter a negative effect. When put together, they indicate that IVF slightly increases the chance of pregnancy in women with unexplained subfertility. However, the authors stopped short of including this in their conclusion due to the small sample sizes, different outcome measures and other limitations of the evidence.

The Human Fertilisation and Embryology Authority (HFEA), the UK regulatory body for fertility clinics, reports the success rates of IVF as ranging from a high of 29% (women aged below 35) to 2% (for women over 44) [17]. However, these data do not take into account spontaneous conception (i.e. they do not indicate how many *extra* births result from IVF), and so only provide half the picture of IVF effectiveness.

If the restriction of IVF to people with obesity is to be justified on the basis of it being insufficiently cost-effective, whilst IVF is still provided to those who are non-obese (by implication, on the basis that it *is* cost-effective in this group) then this should be established through evidence of acceptable quality that establishes the cost-effectiveness of one and the cost-ineffectiveness of the other. The above serves to show that the quality of the evidence relating to effectiveness of IVF in general is very weak.

To assess cost-effectiveness, NICE combines evidence about the effectiveness of an intervention with evaluations of the benefits/harms that result from that intervention, and the cost of providing that intervention. In order to compare across different kinds of healthcare, NICE uses a common currency: the quality adjusted life year (QALY). NICE gathers available evidence about the effectiveness and cost of a given treatment or intervention, and uses this to estimate to what extent that treatment/intervention is likely to increase someone’s length and/or quality of life, and at what cost. One QALY is equivalent to a year of life in perfect health, so if a particular drug extended someone’s life by 15 years and during that time they experienced an average health state of 0.5 (half as good as perfect health) then that drug would create 7.5 QALYs. If that drug cost £10,000 a year then the cost per QALY would be £20,000. Whilst NICE maintains that they do not ‘put a price on human life’ as such, they tend to use a limit of approximately £20–£30 k per QALY for a treatment/intervention to be considered cost-effective [35]. There are various ways in which exceptions can and are made to this limit including, for instance, end-of-life care [19].

NICE’s use of QALYs is somewhat contentious, attracting criticisms relating to both the theoretical possibility of capturing all of the heterogenous benefits (and harms) of a treatment/intervention in a single measure, as well as substantive criticisms of how QALYs over/underweight particular kinds of health and healthcare [39]. On the whole, however, it might be reasonable to accept the flaws associated with QALY use (and seek to ameliorate them) in preference to alternative, less systematic approaches to rationing healthcare resources.

In the context of reproductive technologies, QALYs become much more complicated. In the 2004 guidelines for provision of fertility treatment, NICE states:

There is an important debate about whether the outputs of assisted reproduction can be incorporated into a measure than can be compared with other uses of the same resources. It is not logical to try to derive a quality-adjusted life-year (QALY) measure from live births arising from IVF. [sic]

[31] p. 6.

Despite this hesitancy, NICE subsequently commissioned a report into the economic modelling of QALYs in subfertility treatment. A dubious feature of this modelling is that it only counts the improvements in quality of life post-fertility treatment that attach to the female partner receiving IVF, ignoring both the QALYs gained by creating a life, and those experienced by the recipient’s partner. This is clearly an important decision, since to include the QALYs attaching to the life created would presumably make IVF treatment hugely cost-effective. It might also, however, direct healthcare spending down the path of the repugnant conclusion (i.e. the obligation to create as many people as possible with a minimally decent quality of life, [43]).

The modelling involved in calculating the cost-per-QALY for IVF is complex, needing to take account of the ways in which, for instance, providing treatment at different time points influences the number of QALYs accrued over a lifetime, the varying costs of IVF depending on provider, the potential need for additional healthcare due to pregnancy and childbirth-related complications, and so on [38].^4^ The results of this modelling are presented, showing variations along a number of different dimensions. For instance, the authors calculate how cost-effectiveness varies with maternal age; whether one or two embryos are implanted during treatment; whether it is the first, second or third cycle provided to that individual; and whether the willingness-to-pay threshold is set at a cost-per-QALY of £20 k or £30 k.

The results of such calculations can sometimes be odd, recommending ‘islands’ of treatment (for instance, it is cost-effective to treat 34 year old women with three cycles of IVF using double embryo transfer, but not those aged 35–37, and only those aged 27–33 and 38–41 if the upper cost-per-QALY threshold is used (See figure 14.7 [38]). In translating these calculations into guidelines, NICE must smooth out such oddities. None of the cost-effectiveness calculations published in the National Collaborating Centre for Women’s and Children’s Health/NICE report indicate how obesity is expected to impact the cost-per-QALY of IVF provided to obese women.

This is not to say that there is no way of estimating how obesity could affect the cost-effectiveness of IVF. Papers by Pandey and Bhattacharya [40], Pandey et al. [41], Koning et al. [21], Tremellen et al. [57] and Mahutte et al. [24] all argue that there is insufficient evidence showing the detrimental effect of obesity on the success of IVF compared to non-obese recipients to justify restricting treatment to obese women. This does not mean that obesity has no impact on cost-effectiveness of IVF treatment, nor that weight loss is not a desirable occurrence in obese women prior to pregnancy/IVF treatment. Instead, it indicates that the current policy of commissioners in the UK (and comparable actions of healthcare providers outside the UK) is not based on rigorous assessment of cost-effectiveness and a judgement that whilst IVF provided to non-obese people falls above the threshold for cost-effectiveness, provision to obese people falls below that threshold.

## Proposed Justification III: Something Else

So far, I have considered two potential justifications for excluding obese women from accessing IVF. First, that IVF is futile in obese women, and second, that IVF is insufficiently cost-effective to warrant funding in the context of a healthcare system (such as the NHS) which is required to ensure that a finite budget is spent efficiently. I have rejected these arguments on the basis that they rest on dubious extrapolations from the available evidence. Recall, my claim is not that it is cost-effective to provide IVF to obese women, but that there is insufficient evidence to show that it is significantly less cost-effective than to provide it to non-obese women. Many commissioners in the UK do provide (limited) IVF to non-obese women, but exclude obese women. To claim that evidence relating to the cost-efficiency of IVF allows for fine-grained distinctions between different points on the BMI scale when so little is known about numerous aspects of the effectiveness of IVF (I refer, in particular, to IVF for treating unexplained subfertility) is at best naive, and at worst, intentionally misleading.

I now wish to consider a further possible motivation for excluding obese women from access to IVF. This is the suggestion that those who are responsible for their ill health should be de-prioritised for treatment relative to those who are not responsible for their ill health. My claim will be that it is plausible that judgements of responsibility play an implicit, indirect role in policies which exclude obese people from IVF. I will not argue that this *is* the case since establishing this would require further empirical work. I will comment on whether such a responsibility-based motivation is likely to provide justifying reasons for restricting IVF in this way.

Financial pressure on the UK healthcare system, particularly since 2010, has resulted in changes in the way services are rationed [20]. In 2015, the UK government announced the NHS Spending Review for the next 6 years, which required NHS England to undertake £22 billion in efficiency savings over that period [31]. Unsurprisingly, this has led to a reduction in the provision of services for a number of groups/conditions. Fertility treatment is one area which has seen significant shrinkage in NHS-funded provision. The campaign group Fertility Fairness reported in 2017 that the number of CCGs in England offering the NICE-recommended three cycles of IVF to women who met eligibility criteria halved between 2013 and 2017, going from 24 to 12% of CCGs. They also reported that 23% of CCGs in 2017 offered two cycles; 61% offered one cycle, and 4% (which amounts to seven CCGs) offered no funded IVF at all [9].

IVF may be a relatively easy target for commissioners looking to cut costs by reducing service provision. Evidence is mixed: pro-natal social norms often dominate, resulting in stigma attaching to those who remain childless (both voluntarily and involuntarily) [58, 28, 44], and this might be expected to result in general support for those requiring assistance to have children. There is, however, also evidence that people do not generally think of subfertility as a disease, and this may limit the extent to which treatment for subfertility is seen as a priority for resource allocation within healthcare [28].

Attitudes towards obese people are more decisively negative. Researchers have found extensive evidence of stigma directed towards obese people in a number of contexts, leading to disadvantage. Rebecca Puhl and colleagues have described the pervasive effects of stigmatisation of obesity in a number of contexts, including healthcare settings, the workplace, educational institutions, and interpersonal relationships [45–47]. They report how physicians surveyed associated obesity with poor hygiene, noncompliance, hostility, dishonesty a lack of self-control and laziness. Nurses, dieticians and medical students have also been shown to form negative associations of obese people [45, pp. 792–795]. In the workplace, employers tended to see obese people as less desirable employees; overweight applicants were judged as significantly less neat, productive, ambitious, disciplined and determined; they were assumed to be lazy, less conscientious, less competent, disagreeable and emotionally unstable; they were also thought to have poorer attendance records and to be poor role models. Perhaps as a consequence, obese people tend to earn less, are less likely to be promoted and more likely to get sacked than their non-obese counterparts [45, pp. 789–792]. Children rate obese children as those they would least like to be friends with; obese high school students in a US sample were less likely to be admitted to college than non-obese students with equivalent academic performance; obese students tended to receive less financial support from their families than non-obese students [45, pp. 795–797].

I have argued that the futility and cost-efficiency based justifications for excluding obese people from accessing IVF are weak. I think it is at least plausible, perhaps likely, that such decisions by commissioners are influenced by, first, a need to make savings *somewhere* due to financial constraints, and second, an assessment of public sympathy towards the recipients of certain services/the value of providing those services. Given the highly stigmatised nature of obesity, the ambiguity around the status of subfertility as a ‘disease’, and the confusion around the methodologies used to assess its cost-effectiveness, it is unsurprising that CCGs have chosen to deny IVF treatment to obese people. Fertility treatment may not be an isolated example: a report by the Royal College of Surgeons criticises the restricted provision of elective surgeries (including knee and hip replacements, tonsillectomy and surgeries for snoring, hernia, and varicose veins) for those who are obese or smoke [48].

I take it that excluding some people from healthcare treatment that is available to others on the basis of stigma and moralisation would be impermissible, and contrary to the spirit of the UK system of healthcare provision—informed by procedures such as QALY-assessment—as well as systems of healthcare distribution elsewhere. However, whilst the the Equality Act 2010 outlines protected characteristics which must not form the basis of discrimination (in employment practices, for instance), there is no specific provision made for obesity or, more broadly, physical appearance/attractiveness beyond race, disability or sex [8]. There have been cases of successful legal action for discrimination relating to obesity on the grounds that the individual’s bodyweight constituted a disability, but these are rare and do not reflect either an assumption that obesity generally qualifies as a disability, nor that a legal protection against obesity-based discrimination exists. Similarly, the NHS Constitution repeats a commitment to delivering healthcare that does not discriminate on the basis of those protected characteristics described in the Equality Act 2010. Yet it makes a number of additional commitments based around ‘guiding principles and values’ to, for instance, treat all available to need; meet the highest standards of excellence and professionalism; to put the patient at the heart of NHS activities; to treat people with respect, dignity and compassion [32]. Such commitments do not appear compatible with institutionalised discrimination and stigmatisation of people with obesity. Thus, if part of the explanation for why obese people are excluded from accessing NHS funded IVF treatment is that they are a ‘soft target’ for cost cutting, there would be reason to object to the behaviour of commissioners complicit in this systematic discrimination. I cannot establish that this is the case here, but have instead sought to show that the evidentiary basis of this policy is weak, that healthcare commissioners are under severe financial pressures, and that both fertility treatment and obese people may be publicly unpopular.

Some might argue that there *is* a relevant difference between those who seek IVF and who are a healthy weight, and those who are obese. In the next section I will briefly consider whether responsibility might ground a justification for excluding obese people from accessing NHS-funded IVF.

## Responsibility, Obesity and Subfertility

It is a widely shared intuition that the consequences of people’s behaviour should track responsibility, although questions about what exactly responsibility requires, and how it relates to free will and determinism remain disputed [36, 54]. This can reflect both benefits and harms: people should *reap the rewards* as well as *bear the burdens* of their behaviour. Responsibility is a perfectly normal feature of many areas of life, including personal/social relationships, criminal justice policy, employment practices, sporting achievements, and so on.

The requirements and implications of responsibility can be analysed in a number of ways. A common distinction is drawn between *causal* and *moral* responsibility, with the former identifying the causal role an actor played in bringing about some consequence, and the latter typically identifying a deeper relationship between an agent and a consequence, often resulting in responses such as praise or blame. It is the presence or absence of moral responsibility for some consequence that is often used to determine how it is appropriate to treat an agent when we seek to ‘hold her responsible’.

It is common to identify two broad conditions for moral responsibility: the epistemic and control conditions. The first requires that the agent could foresee the (likely) consequences of her actions; she understood how her behaviour would impact the world. The second requires that the agent be able to control her actions; she could have behaved differently. Some version of these conditions has been incorporated into most accounts of moral responsibility since Aristotle, though the exact demands (what counts as sufficient understanding? How much control must the agent have had?) vary [1, 12].

It is not clear how we should go about developing a systematic means of assessing whether or not an individual was responsible for some behaviour. There are, however, cases which bookend the spectrum of responsible/not responsible. For instance, if I am sitting quietly reading a book when a remarkably strong gust of wind dislodges me from my chair and causes me to collide with you, injuring your arm, it seems clear I should not be judged morally responsible (or blameworthy) for your broken arm. I was not an active agent in this event, and we should not call my colliding with you an ‘action’ of mine (some philosophers prefer to call these non-agential movements ‘mere happenings’ [13]). In contrast, if, upon spotting you (my arch nemesis), I discard my book and leap from my chair, and purposefully attack you with the full intention of breaking your arm, then (assuming I am not experiencing some kind of psychotic episode, or sleep walking, or under the influence of mind-altering substances, etc.) it seems clear that I am morally responsible (and blameworthy) for injuring you.

Turning to the current case, the challenge is to consider whether or not those who are obese and subfertile should be considered morally responsible for their subfertility (and further, whether this has any relevance to the provision of fertility services). Evidence suggests that awareness of the impact of obesity on fertility is limited (e.g. [5, 4]), though that does not mean all of those who are obese and subfertile were unaware of the fertility-related risks of obesity. Intuitively, obesity seems under individual control, however evidence from diverse research programs including work identifying the social determinants of health and the psychology of behavioural control suggests it is often extremely difficult for individuals to make changes to their lifestyles that result in significant weight loss [25, 26, 53]. Further, it will often be unclear whether a particular individual who is both obese and subfertile is subfertile *because* of her obesity. The causal role of obesity will be underdetermined at an individual level.

It is beyond the scope of this paper to make any kind of estimate as to the proportion of people who are both subfertile and obese who sufficiently fulfil the epistemic and control conditions of responsibility to be considered morally responsible for their subfertility. However, it seems reasonable to assume that there exist both people with subfertility who were oblivious to the effect of obesity on fertility/lacked control over their obesity, and people with subfertility who were aware of the effect of obesity on fertility/possessed control over their obesity. Thus, in the population of obese people with subfertility there will be some mix of morally responsible and not morally responsible individuals.

If responsibility were to be incorporated into decisions about IVF provision, it would be necessary to distinguish between those who were morally responsible for their obesity-linked subfertility and those who were not. This would require delving into individual cases of subfertility and seeking a way of determining who is and is not responsible for their subfertility. As well as being highly intrusive (and potentially impermissible) this would likely be financially costly. What would be the pay-offs of such an approach?

One possibility is that rationing IVF in this way would act as an incentive for people to maintain a healthy BMI (a desirable outcome so far as health promoters are concerned). It has been proposed by some that educating young people about the damaging effects obesity could have on their fertility is a potential tool for health promotion and weight loss [22]. The pay-off of this effect would be dependent on whether or not excluding obese people from IVF resulted in fewer people becoming obese/obese people losing weight. To my knowledge, no research has estimated the effect of BMI-restricted IVF policies adopted by commissioners in the UK on obesity, and from what is known about people’s attempts to lose weight, it seems unlikely that denial of NHS-funded IVF treatment for obese women would have a significant positive effect on weight loss efforts [11].

Another consequentialist justification for BMI-restricted IVF is that it reduces the number of people eligible for treatment and thus saves money. However, whilst discussions of healthcare allocation may often emphasise cost-savings, this should not be taken out of context. The reason for saving costs on some areas of healthcare is so that those resources can be more effectively used in other areas of healthcare, improving the cost-efficiency of overall provision. If obese people can benefit from IVF (and the money spent on IVF would not create more benefits if it were spent on some other kind of healthcare) then it makes sense to provide IVF to obese people. Another drawback of such a justification would be that it seems to arbitrarily limit some people’s access to healthcare.

Finally, justice based arguments might motivate BMI-restricted IVF provision amongst those responsible for their subfertility. The most demanding version of this claim relates to desert: that it is a *good thing* in and of itself if people get what they deserve. Translated into the subfertility case, this claim would imply that it is good for people who are responsible for their obesity-linked subfertility to be excluded from treatment, and bad for them to receive treatment (with the reverse being true for those not responsible for their subfertility). A weaker defence of BMI-restricted state funded IVF policies would be broadly luck egalitarian (see for example [50]). This, roughly, assumes that as a matter of justice, the state is obligated to assist those who experience bad brute luck but not those who experience bad option luck (i.e. the state is only obligated to support those who experience certain kinds of disadvantage that results from factors for which they were not responsible). Luck egalitarians may still assist people who experience bad option luck (i.e. those responsible for their misfortune), but this is not a requirement of justice, and if it involves failing to assist someone with bad brute luck then it will not be permissible. Essentially, luck egalitarianism could support a policy of prioritising treatment for those whose subfertility resulted from factors for which they were not responsible.

Ultimately, it must be considered whether such justifications for seeking to distinguish between those who are and are not responsible for their obesity-linked subfertility are sufficiently powerful to overcome the reasons for not drawing such distinctions. As mentioned, countervailing reasons could point to the difficulty of making such judgements and the likely intrusiveness involved; the danger of further stigmatising certain disadvantaged groups; and principled objections that healthcare providers just should not take account of responsibility when making allocation decisions.

## Conclusion

In this paper, I have advocated a sceptical stance towards ‘evidence-based’ justifications for excluding obese people from NHS-funded IVF treatment. I have argued that the lack of high quality evidence regarding the effectiveness of IVF in both obese and non-obese populations, and the confusion around how QALYs are calculated in relation to the outcomes of IVF, give us reason to be hesitant in accepting the claim that this policy is justified on cost-efficiency grounds. Whilst ensuring the efficient provision of healthcare is an important function of the state, methodologies such as evidence synthesis and QALY calculations must be applied with full acknowledgement of the degree to which these techniques incorporate numerous controversial assumptions and rough estimations and often must extrapolate from weak evidence. Claims that the evidence is of sufficient clarity to make decisive cost-effectiveness cutoffs appropriate is disingenuous. Rather, I propose that references to the cost-inefficiency of providing IVF to obese people may mask other influences, such as negative attitudes towards obese people that render them easy targets for cost-cutting commissioners. Finally, I introduced an alternative approach to justifying policies which exclude obese people from accessing IVF, based on judgements of responsibility in addition to other factors (such as the defence of a luck egalitarian approach to healthcare provision). Whilst this discussion has been kept necessarily brief, my suspicion is that such justifications would ultimately struggle to support a policy which required intrusive investigation into people’s disease aetiology and judgements of responsibility.

## Footnotes

1 These data have been collected by the group Fertility Fairness which used freedom of information requests to access individual commissioning organisations’ policies regarding BMI and IVF provision. Such policies do not guarantee that no person with a BMI outside this range receives NHS funded IVF since there remains the possibility for individual physicians to circumvent the decisions made by commissioners.

2 Even this may not be wholly uncontentious. Evidence suggests people derive value from being provided with ART even when it *reduces* their chances of conception, as in a paper by Bhattacharya et al. [2] where satisfaction with treatment was higher in the treatment group (who received clomifene citrate) than in those in the expectant management group, even though it turned out that clomifene citrate acted as a mild contraceptive.

3 Cochrane reviews synthesise data from separate studies and frequently provide a meta-analysis of the evidence from separate research trials in order to try to better assess the effect of a particular intervention.

4 For more on the methods used to calculate QALYs for fertility-affecting treatment, see [15, 49].
